# Taphonomic bias of hydrothermal silicification in biodiversity patterns of Cambrian shelly pavements from the Iberian Chains, NE Spain

**DOI:** 10.1038/s41598-025-01878-3

**Published:** 2025-05-14

**Authors:** Blanca Martínez-Benítez, Jorge Esteve, J. Javier Álvaro

**Affiliations:** 1 Instituto de Geociencias (CSIC-UCM), Dr. Severo Ochoa 7, 28040 Madrid, Spain; 2 Facultad de Ciencias Geológicas, Universidad Complutense, José Antonio Nováis 12, 28040 Madrid, Spain

**Keywords:** Hydrothermalism, Silica, Taphonomic bias, Microfossil, Cambrian, Biogeochemistry, Environmental sciences, Solid Earth sciences

## Abstract

Silicified fossils are ideal for taxonomic studies because they can be extracted without damage using acids. However, permineralization commonly introduces taphonomic biases: as silica neomorphism occurs, silicified fossils are also susceptible to loss of microstructural fidelity and diagnostic characters useful for taxonomic determination. These processes are studied in the traditional ‘lower‒middle Cambrian’ transition of the Iberian Chains, where the macrofossil content includes variable abundances of trilobites and linguliformean brachiopods. In contrast, acid etching of partly silicified limestone interbeds offers a quite different biodiversity pattern, dominated by trilobite larvae, chancelloriids and sponge spicules, accompanied by locally abundant calcite- and phosphate-walled brachiopods, echinoderm holdfasts, and psammosphaerids and serpulids encrusting disarticulated sclerites. Petrographic, geochemical (chondrite-normalized REE, gull-wing patterns), cathodoluminescence (prominent yellow emission bands between 560 and 580 nm, and weaker blue bands between 440 and 500 nm) and Raman spectral data (main peak at 465.2 cm^−1^ and secondary ones at 205.5 and 128.1 cm^−1^) yielded by silicified microfossils reveal that quartz precipitation was induced by a distinct episode of acidic hydrothermal activity, close to 100 °C. The event is linked to a broadly penecontemporaneous tectonic breakdown event, where fissures served as conduits for silica fluids.

## Introduction

A Lagerstätte (German for ‘storage place’^1^ is a fossiliferous outcrop displaying unusual preservation patterns. The factors that controlled such special taphonomic conditions include, among others, stagnation (eutrophic anoxia), obruption (rapid burial), microbial sealing, salinization and permineralization (authigenic cementation) processes^2^. Permineralization by silicification is one of the preservation pathways through which both non-biomineralizing structures and carbonate skeletal material can be three-dimensionally replicated. In some exceptional examples, recognized as Konservat-Lagerstätten, the casts are encrusted by silicified cyanobacterial films and mats (‘death mask’ preservation^3,4^. Silicified fossils are ideal for taxonomic studies because they can be extracted without damage by acid etching. However, silicification also currently introduces severe taphonomic biases because silicified fossils are susceptible to loss of ultra- and microstructural fidelity as silica neomorphism occurs. After precipitation, silica goes through a series of diagenetic transformations: opal-A (amorphous) > opal A’ (secondary) > opal CT > opal CT (reordered phase) > cryptocrystalline quartz or chalcedony > micro- and macrocrystalline quartz^5^. As a result, the increasingly coarse nature of the quartz and chalcedony crystals necessarily obliterates the diagnostic characters of original skeletons for taxonomic determination.

The limestone interbeds of the Valdemiedes Formation in the eastern Iberian Chain, NE Spain, preserve the permineralized remains of a diverse microfossil assemblage, which comprises silicified trilobite larvae^6^, sponge spicules and chancelloriid sclerites^7^, echinoderm holdfasts^8^, linguliformean brachiopod valves, and psammosphaerids and serpulids encrusting disarticulated sclerites^9^. These shelly pavements do not represent a Konservat-Lagerstätte because their skeletal ultrastructures and textures are obliterated by growth of macroquartz (> 20 μm) crystal mosaics. Acid etching of these partly silicified limestone strata allowed the identification of higher biodiversity patterns than those yielded by interbedded shales and non-silicified limestones, where acid etching is not effective. However, their preservation as mosaics of macroquartz commonly precludes their taxonomic assignment at the species level. Their taxa have been commonly left in open nomenclature or assigned to a generic level, emphasizing that taxonomic identification was not fully accomplished.

The aim of this work is to ascertain the origin of the silica permineralization recorded in some limestone strata of the Valdemiedes Formation in the eastern Iberian Chain. A discussion about the involved processes of silica precipitation is included as a taphonomic bias in estimation of Cambrian biodiversity patterns.

## Geological, stratigraphic and environmental overview

**Fig. 1:**
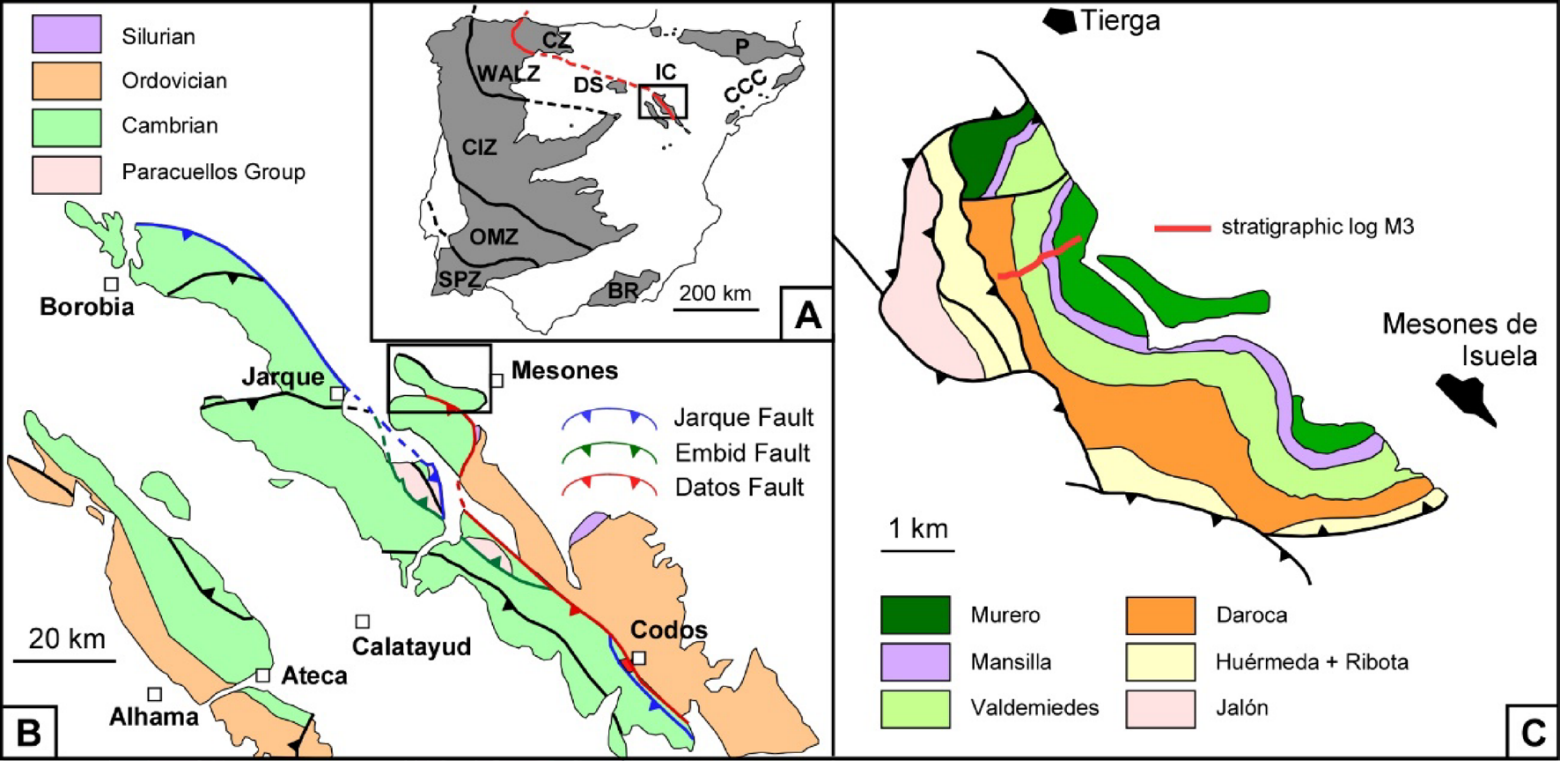
Geological setting of the study area. (**A**) Pre‒Variscan exposures of the Iberian Peninsula (in grey); tectonostratigraphic abbreviations: BR, Betic Ranges; CCC, Catalan Coastal Chain; CIZ, Central Iberian Zone; CZ, Cantabrian Zone; DS, Demanda Sierra; IC, Iberian Chains; OMZ, Ossa-Morena Zone; P, Pyrenees; SPZ, South Portuguese Zone; WALZ, West Asturian-Leonese Zone; red line is connecting WALZ/CZ contact throughout DS and IC. (**B**) Geological sketch of the pre‒Variscan outcrops in the northern Iberian Chains (boxed in A). (**C**) Geological map of the Mesones de Isuela-Tierga unit in the northeastern edge of the eastern Iberian Chain; adapted from^9,10^.

**Fig. 2:**
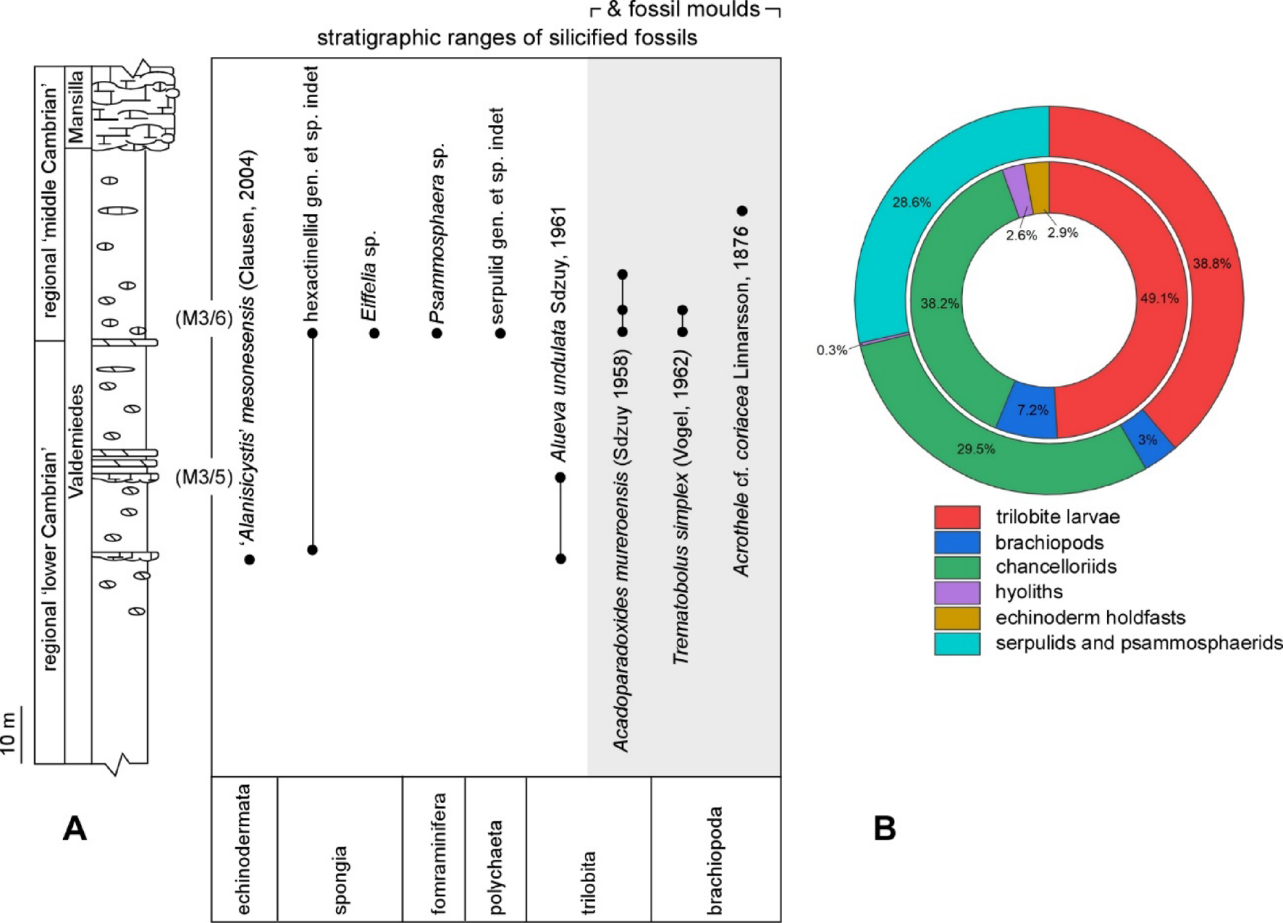
(**A**) Stratigraphic sketch of the Cambrian Epoch 2‒Miaolingian (traditional ‘lower‒middle Cambrian’) transition in the Iberian Chains, with stratigraphic ranges of sampled taxa. (**B**) Palaeodiversity values (*n* = 100) of the M3/5 (inner ring) and M3/6 (outer ring) limestone interbeds of the Valdemiedes Formation in the vicinity of Mesones de Isuela village^8,14–17^.

The Iberian Chains of NE Spain comprise two NW‒SE trending Palaeozoic inliers surrounded by Mesozoic and Cenozoic ranges (Fig. 1A‒B). They represent the lateral prolongation of two Variscan tectonostratigraphic units from NW Spain, the Cantabrian and West Asturian-Leonese zones of the Iberian massif^10^. Cambrian strata in the Iberian Chains are dominated by siliciclastic deposits, punctuated by two mixed (carbonate-siliciclastic) intervals. One of them is represented by the Mesones Group, which comprises the Cambrian Series 2‒Miaolingian (traditional ‘lower‒middle Cambrian’) transition (Fig. 2). The group has been subdivided into three units, named, from bottom to top, the Valdemiedes, Mansilla and Murero formations^11^. The Valdemiedes Formation, on which this work is focused, is 20–150 m thick and consists of shale/carbonate alternations. The carbonate interbeds are beige limestones or yellowish dolostones, with stromatolitic or bioclastic textures. In the vicinity of Mesones de Isuela village (Fig. 1C), several limestone interbeds crossing the regional ‘lower‒middle Cambrian’ boundary interval^12^ are bioclastic packstones to wackestones. Some sedimentary cycles, up to 4 cm thick, display single to amalgamated, scoured bases (with local gutter and tool marks) capped by graded shell layers, in some cases displaying fining and low-angle laminae. Shelly fossils are wholly disarticulated and fragmented at the bottom, whereas the top of the cycles can display partly articulated sclerites, in some cases up to 12 cm in size (paradoxidid sclerites). These structures have been interpreted as the result of alternating storm-induced events interrupting fair-weather wave conditions^13^.

The conformably overlying Mansilla Formation, 10–70 m thick, shows significant lateral modifications in thickness and facies. The unit consists of bedded to nodular limestone/shale alternations, purple to reddish in colour. The lower thicker carbonate interbeds, up to 1.2 m thick, comprise amalgamated bioclastic packstone to wackestone textures, similar to those reported for the Valdemiedes Formation. In contrast, the thinner carbonate layers and nodules from the upper part show wackestone-to-mudstone textures with preservation of partly articulated trilobites and brachiopods. Sedimentation of this characteristic facies association, named ‘griotte facies’ by their purple to reddish colours in SW Europe and Morocco^18^, reflects the interaction of two factors: episodes of tectonic activity interacting with cycles of Milankovich-like orbital forcing. A quantitative analysis of the former revealed that several tilting disturbances are recorded in the carbonate/couplets of the Valdemiedes and Mansilla formations, with its most significant tectonic event marking the Valdemiedes/Mansilla contact^19^. Complex and varied subsidence patterns across a horst-and-graben palaeorelief recorded stepwise tectonically induced disturbances. In addition, a multifold cyclicity of these carbonate/shale cycles has been studied by spectral analyses. Their high-frequency cycle-stacking patterns were interpreted as a two-fold hierarchical interaction, where the carbonate/shale alternations would be linked to the precessional/obliquity ratio predicted for early Palaeozoic times^20^.

## Results

### Early diagenetic processes

**Fig. 3:**
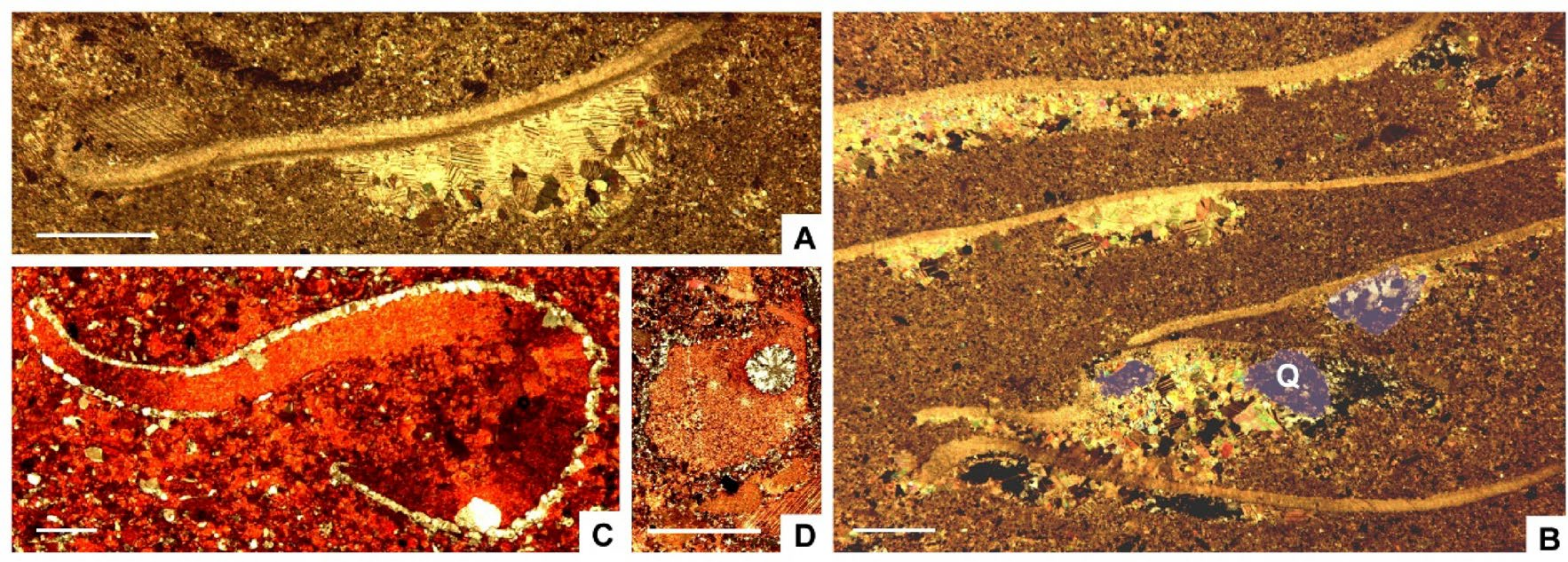
Thin-section photomicrographs of the bioclastic limestones from the Valdemiedes Formation crossing the traditional ‘lower‒middle Cambrian’ transition in the vicinity of Mesones de Isuela, eastern Iberian Chain. (**A**) Shelter pore underlying a trilobite sclerite progressively occluded with bladed and equant calcite crystals. (**B**) Bioclastic wackestone with different trilobite sclerites capping shelter pores occluded with calcite, and subsequently crosscut by vuggy pores infilled with mosaics of quartz (Q). (**C**) Limestone stained with Alizarine red S, emphasizing the difference between a calcitic trilobite sclerite and a quartz veneer encrusting its skeletal wall. (**D**) Stained echinoderm ossicle including a subcircular vug occluded with chalcedony; all scale bars = 1 mm.

**Fig. 4:**
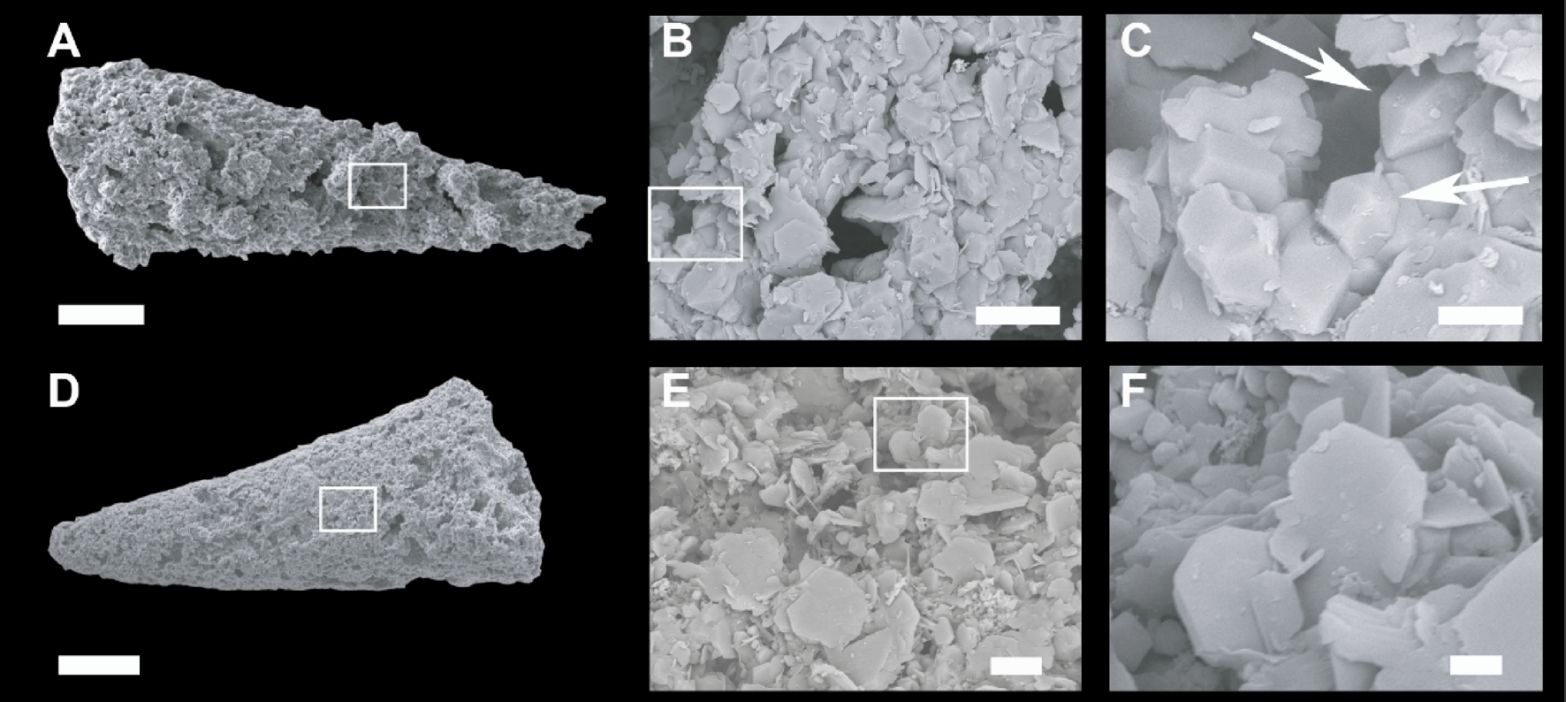
Hyolith steinkerns rich in clusters of quartz and chlorite crystals. (**A**) Steinkern composed of chlorite-quartz clusters. (**B**) Detail (boxed area) of quartz cluster with euhedral pyramidal quartzs. (**C**) Detail of pyramidal quartzs (arrowed). (**D**) Steinkern rich in Fe-rich chlorite flakes (**E**) arranged in euhedral platy crystals with sub-hexagonal habits (**F**). Scale bars: A = 200 μm, B = 20 μm, C = 5 μm, D = 200 μm, E = 10 μm, F = 2 μm.

**Fig. 5:**
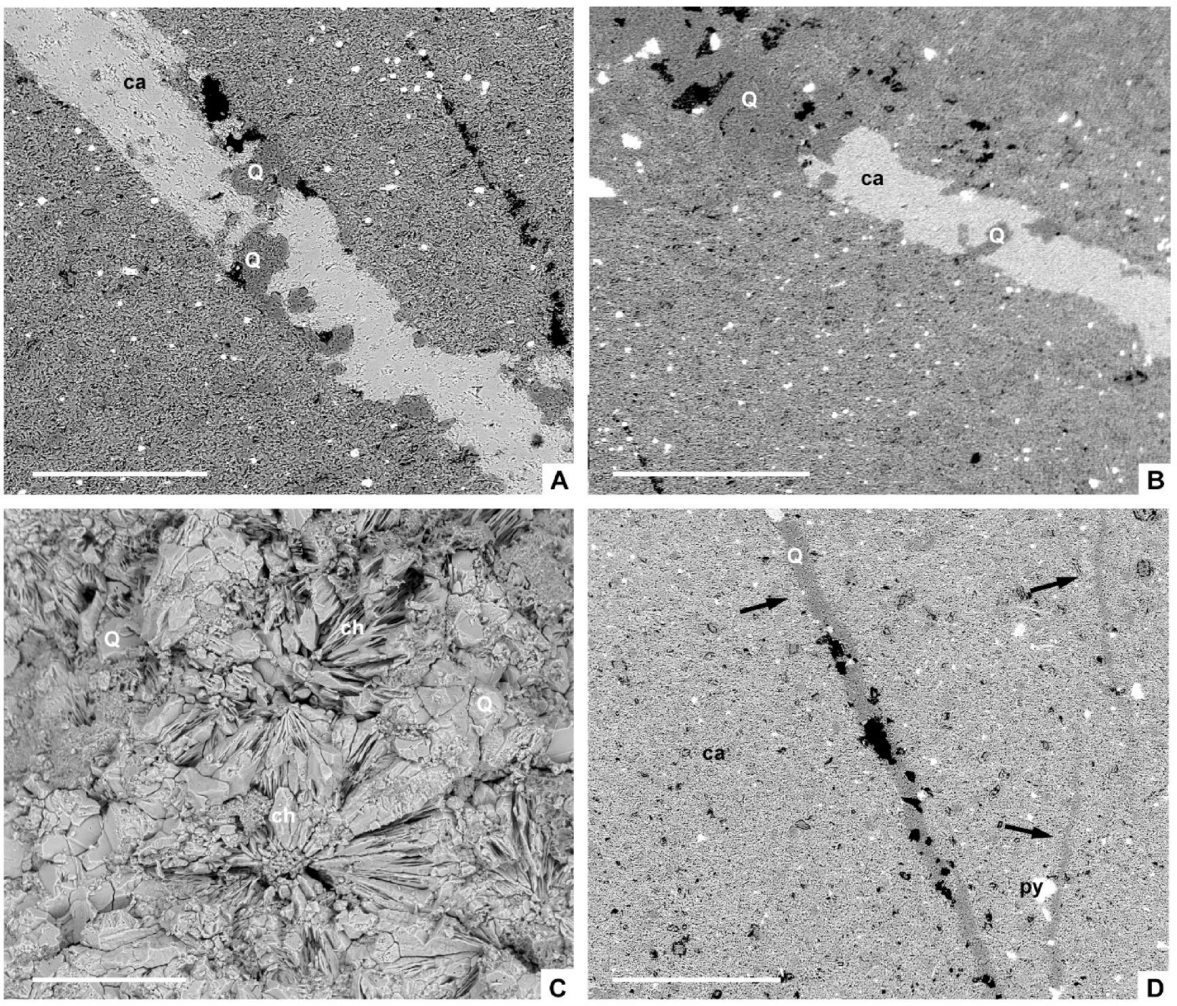
SEM photomicrographs of silica replacements and precipitates recorded in the Valdemiedes Formation of Mesones de Isuela, eastern Iberian Chains. (**A**) Detail of trilobite sclerite, embedded in a calcite matrix mixed with silt-sized quartz and clay minerals, showing a partial silicification that emphasizes its outline. (**B**) Detail of another trilobite sclerite with partial to complete silicification. (**C**) Detail of a silica mosaic with chalcedony rosettes and quartz in the broken surface of a sample. (**D**) Fissure (arrowed)-affected host limestone with secondary porosity occluded with quartz. Abbreviations: ca, calcite; ch, chalcedony; py, pyrite; Q, quartz. Scale bars = 300 μm (**A**), 500 μm (**B**, **D**) and 300 μm (**C**).

The partial silicification of limestone interbeds from the Valdemiedes Formation is absent in the overlying Mansilla Formation. In the Valdemiedes limestone strata, original porosity observed in thin-section includes intragranular (mainly bioclastic), intergranular and shelter pores, whereas secondary porosity predating late-diagenetic stylolites comprises vuggy networks interconnected by fissures. Primary porosity is commonly occluded by syntaxial overgrowths of calcite cement on echinoderm ossicles, as well as drusy calcite cements. Drusy cements, mainly occluding shelter pores underneath skeletal fragments (Fig. 3A‒B), developed: (i) isopachous bladed (elongated crystals oriented perpendicular to the skeletal surface, and with euhedral terminations into the pore space), up to 400 μm long and 100 μm wide; and (ii) equant and blocky calcite cements, up to 100 μm in size, filling the remaining pore space. Solution vugs, up to 1.4 mm in size, are non-fabric selective (Fig. 3B). They are subrounded to irregular in shape, and their global porosity can average about 10‒15% of rock volume. Skeletons are partly replaced by the same cements that occlude vuggy pores: mosaics of microquartz (< 20 μm in size) to macroquartz (> 20 μm) (Figs. 3C, 4A–C and 5A‒B) and chalcedony (Fig. 3D); chalcedony crystals, about 50–100 μm long, are arranged as radiating spherulitic textures (Fig. 5C). Similar veneers of microquartz, up to 80 μm thick, also occur encrusting some skeletons (Fig. 3C), enabling their extraction after acid etching. Fe-rich chlorites are ubiquitous and occur as disseminated flakes within the matrix and honeycomb to boxwork (crystals tilted to transverse to microfossil walls) arrangements of euhedral platy crystals with pseudo-hexagonal habits (Fig. 4D–F).

Some of the above-reported vuggy pores occluded with silica are associated with disseminated veins and cracks, also infilled with micro- and macroquartz (Fig. 5D). Veinlets are locally arranged following fissure networks that disappear at the Valdemides/Mansilla contact. Veins, up to 3 cm long and 1 cm wide, are composed of multiple quartz generations, with common dissolution and overlapping textures. Quartz crystals are blocky elongated crystals surrounded by subhedral quartz grains located close to the centre of the veins. Fluid inclusions are absent or less than 1 μm in size.

### Rare earth element (REE) geochemistry of quartz

Silicified microfossils (4 g) were extracted after acetic acid etching from two levels: M3/5 (top of regional ‘lower Cambrian’) and M3/6 (base of regional ‘middle Cambrian’; Fig. 2). The REE + Y elements of the quartz mosaics analysed by Inductively Coupled Plasma Mass Spectrometry (ICP-MS) in the Complutense University of Madrid can be found in Table 1. The analyzed mixture of microquartz, macroquartz and chalcedony shows moderate to high ΣREE + Y concentrations ranging from 128.2 to 216.1 ppm. Light rare earth element (LREE) concentrations range from 109.1 to 189.6 ppm (average value is 148.9 ppm), and heavy rare earth element (HREE) concentrations from 9.5 to 13.0 ppm (average 11.1 ppm). The LREE/HREE ratio ranges from 11.4 to 14.9 emphasizing a distinct enrichment in LREE. In addition, the Y/Ho values range from 25.2 to 28.3 (average value is 26.7), the La/Ho ones from 62.8 to 80.0, and the Sm/Nd ones from 0.16 to 0.19.

**Table 1:** Chemical composition of REE (in ppm) from silica cements in stratigraphic levels M3/5 (top of regional ‘lower Cambrian’) and M3/6 (base of regional ‘middle Cambrian’).

	La	Ce	Pr	Nd	Sm	Eu	Gd	Tb	Dy	Y	Ho	Er	Tm	Yb	Lu
M3/5_1	24	53	6.7	27	5.1	1.1	4.3	0.47	2.2	9.9	0.35	0.73	0.086	0.53	0.082
M3/5_2	22	51	6.3	25	4.8	1.07	4.2	0.45	2.1	9.5	0.35	0.73	0.086	0.52	0.079
M3/5_3	24	55	6.6	26	4.8	1.08	4.3	0.46	2.2	9.8	0.35	0.73	0.086	0.54	0.08
M3/5_4	23	53	6.3	25	4.7	1.08	4.1	0.46	2.1	9.6	0.34	0.72	0.084	0.55	0.077
M3/6_1	39	89	9.6	37	6	0.99	4.4	0.5	2.7	12.7	0.5	1.26	0.19	1.34	0.21
M3/6_2	40	92	10	38	6.3	1.05	4.7	0.53	2.8	13.1	0.52	1.3	0.19	1.35	0.21
M3/6_3	40	87	10	38	6.4	1.04	4.5	0.5	2.7	12.8	0.5	1.27	0.19	1.37	0.21
M3/6_4	41	94	10	38	6.4	1.07	4.9	0.53	2.9	13.5	0.53	1.26	0.19	1.43	0.22

**Fig. 6:**
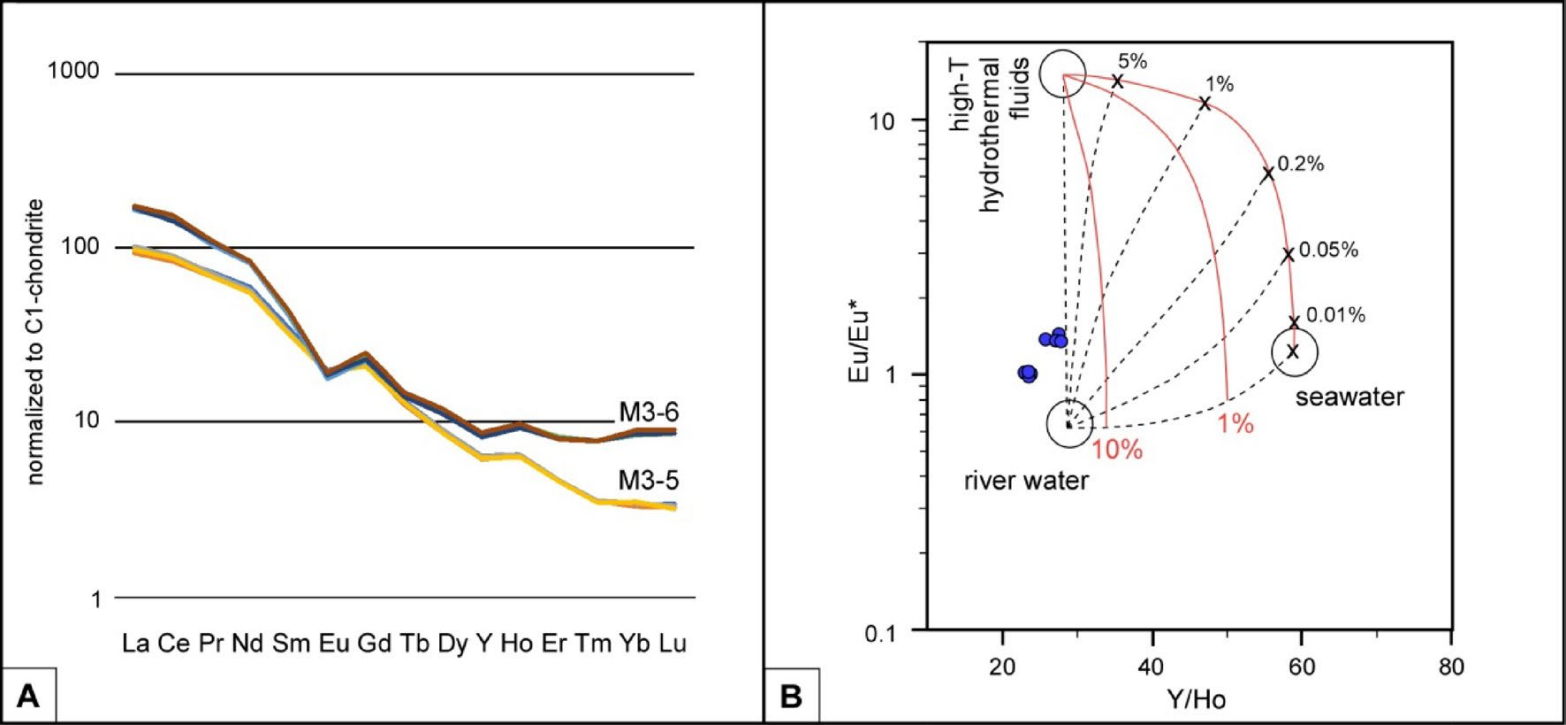
REE patterns of silicified microfossils. (**A**) C1 chondrite-normalized REE distribution of samples M3/5 and M3/6 showing a distinct gull-wing pattern. (**B**) Bivariate plot diagram showing the relationship between Y/Ho and Eu/Eu* (normalized to C1 chondrite) ratios, indicating the lack of high-T hydrothermal, river and seawater influence^22,23^.

The REE patterns of the samples normalized to the C1-chrondrite^21^ standard (Fig. 6A) show a distinct decline from La to Eu, with Pr_(SN)_/Yb_(SN)_ ratios = 12.4‒21.9. Ce anomalies are not significant, with Ce/Ce* ratios ranging from 0.98 to 1.1. A negative anomaly in Eu is distinct, with Eu/Eu* values ranging from 0.56 to 0.73 (Fig. 6A).

### Raman and cathodoluminescence spectra of quartz

**Fig. 7:**
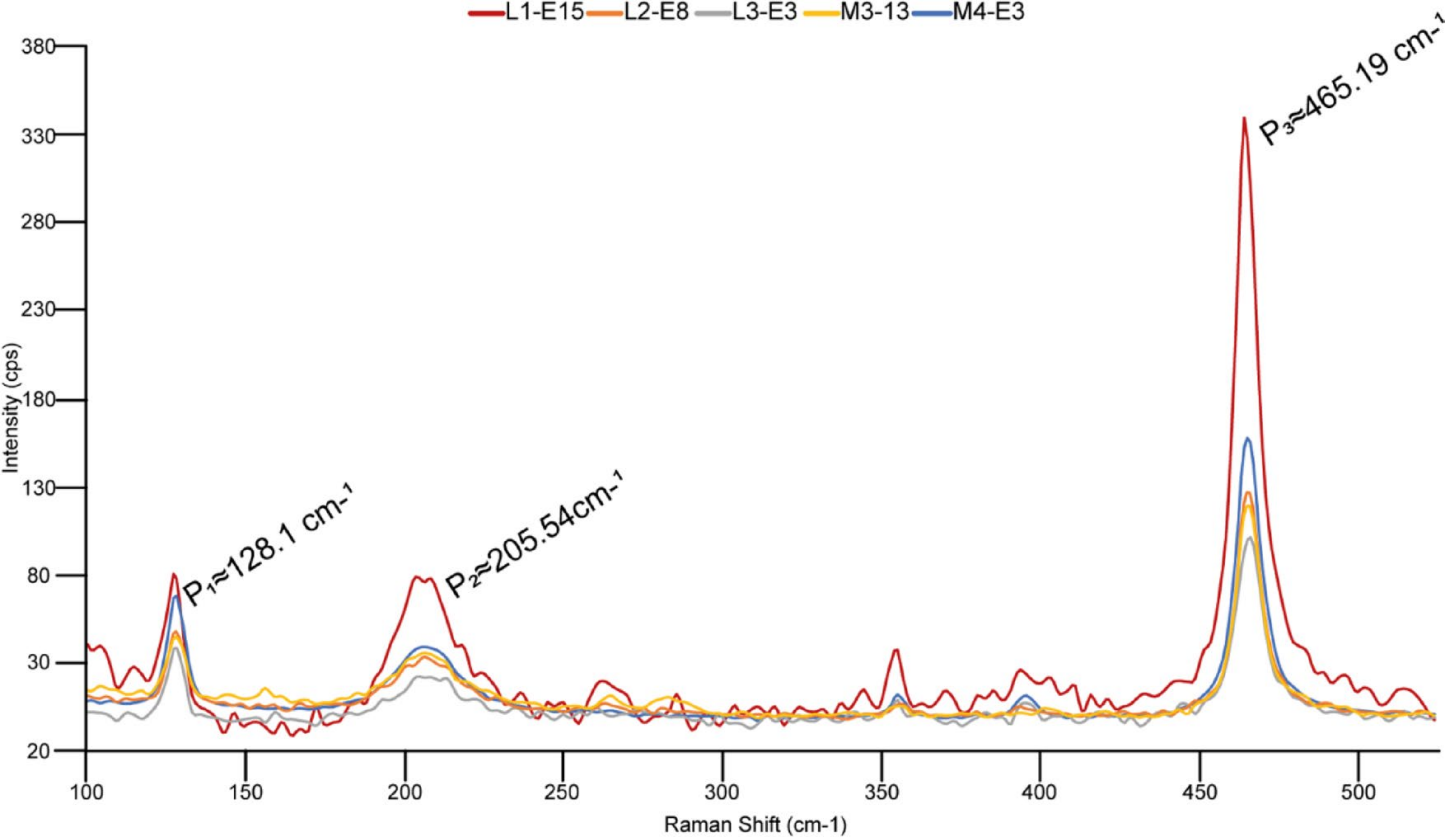
Raman spectra of five 10 μm-long transects analyzed from hydrothermal micro- and megaquartz mosaics of the Valdemiedes Formation, characterized by peaks at 128.10, 205.54 and 465.19 cm^‒1^.

**Fig. 8:**
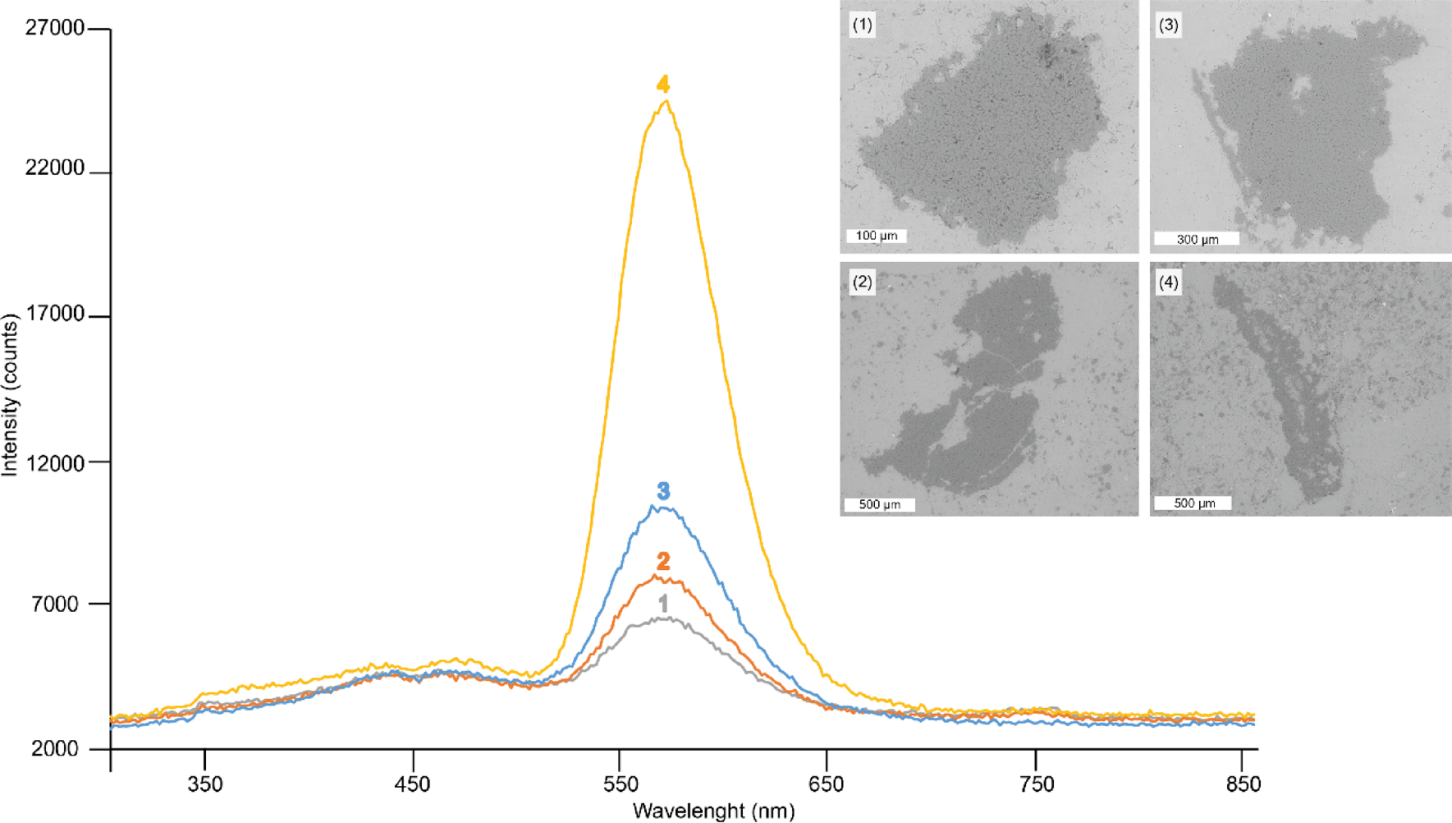
CL emission spectra of four 10 μm-long transects analyzed in hydrothermal megaquartz mosaics (1 to 3) and microquartz veneers (4) of the Valdemiedes Formation, with peaks at ca. 570 nm. Analyzed spots are illustrated by their SEM photomicrographs. Different intensities of CL emission bands reflect radiation by different quartz lattice damages.

Several Raman spectra were acquired from the aforementioned micro- and megaquartz crystals at room temperature. They display a strong peak at 465.19 cm^−1^ and two subsidiary ones at 128.1 and 205.54 cm^−1^ (Fig. 7). Their cathodoluminescence (CL) spectra show a prominent yellow emission band, ranging from 560 to 580 nm (with a maximum at 570 nm), and two weak blue emission bands between 440 and 480‒500 nm (Fig. 8). SEM-EDS analysis revealed that quartz crystals include variable amounts of Al (Si/Al ratio ranging from 120 to 260) and K (Si/K ratio from 200 to 220).

## Discussion

### REE geochemical composition

REE composition of silicified microfossils composed of micro- and macroquartz and chalcedony shows distinct fractionation, with enrichment in LREE and depletion in HREE. The shape of the chondrite-normalized patterns exhibits a typical gull-wing outline^24^ (Fig. 6A), with a small but noticeable negative Eu anomaly, and light (La–Sm) and heavy (Gd–Lu) ‘wings’. This pattern points to distinct water-rock interaction processes under extreme reducing conditions^25,26^, which keep the Eu^2+^ with stability in the host rock, whereas Eu^3+^ is more susceptible to be leached into the surrounding fluid^27^. The REE gull-wing signature is characteristic of shallow acid-sulfate geothermal and alkaline and chloride-dominated waters systems, and their hydrothermal precipitates in encasing host-rocks. The negative anomaly in Eu would suggest both plagioclase fractionation, in the acidic source rocks, and relative intensity in the alteration of plagioclase. Geochemical gull-wing patterns have been reported, among other places, in geothermal areas of Japan^23^, New Zealand^25^, Italy^27,28^, Mexico (El Chichón volcano^29,30^, Iran^26,31^, and the Azores Archipelago^32^.

In addition, Y/Ho ratios represent chondritic to slightly super-chondritic values. A bivariate diagram of Y/Ho vs. Eu/Eu* ratios^22,23^ illustrate values plotting outside the triangle marked by high-temperature hydrothermal, river and seawater fluids (Fig. 6B). In addition, Y/Ho and Eu/Eu* ratios show a positive linear correlation (r^2^ = 0.921). The gull-wing pattern, reported Y/Ho ratios and weak negative Eu anomalies suggest silica precipitation under low-temperature acidic hydrothermal waters interacting with silica-rich host rocks^25,33,34^, such as the sandstone and shale interbeds of the Valdemiedes and underlying strata.

Based on the chemical data measured from modern hydrothermal fluids sampled in present-day rifting, drifting and back-arc basins^35,36^, a negative correlation between vent temperatures and Eu/Eu* values was established as a proxy for hydrothermal systems. The relationship is expressed as Eu/Eu* = −0.123 × T + 49.604 (with r^2^ = 0.864), where T is the temperature (in Kelvin) of hydrothermal fluids. According to the Eu/Eu* values calculated for the hydrothermal quartz encased in the Valdemiedes Formation, the temperature of precipitation would range from 124.2 to 124.8 °C.

### Raman spectra

Among the Raman spectra acquired from micro- and megaquartz crystals at room temperature, α-quartz is the most stable and common polymorph of silica. Its main shift located at 464 cm^−1^ has been interpreted as the result of bending vibrations of the intratetrahedral O‒Si‒O angles^37^, whereas the lattice instability of quartz is expressed by subsidiary peaks at 128 cm^−1^ (Si‒O‒Si bending vibration) and 206 cm^−1^ (SiO_4_ tetrahedral vibrations)^38,39^. The linewidths of the three peaks display strong temperature dependence: e.g., the 464 cm^−1^ mode broadens from 8 cm^−1^ at room temperature to about 40 cm^−1^ at 800 °C^40^. These uncertainties can be mistakenly confused with the Raman peaks of other silica polymorphs: e.g., moganite, relatively common in hydrothermal environments, shows a distinct band at 463 cm^−1^, which can be linked to the characteristic 465 cm^−1^ peak of α-quartz^41^.

Although Raman spectroscopy is usually assumed as a non-destructive technique, concentrated laser spots can generate localized heating leading to crystalline changes, devolatisation, and even melting of the sample^42^. As a result, the heating effects somewhat drift the Raman band shifts. A monitorization of the peak position can be determined with the standard Raman palaeothermometry method^43^. Several features of Raman spectra, such as peak position, linewidth and intensity, are modified by temperature changes and can therefore be used as a thermometer^44^. To reduce uncertainty in the peak position of the 465.19 cm^−1^ peak (Fig. 7), the collected spectra were fit to a Voigt function using a Python script. Using Hibbert et al.^42^ method, the dataset shaping the curve was customized by the equation *y* = ‒0.01*x* + 464.81, where *y* is the peak position and *x* is the corresponding temperature in °C, with a coefficient of determination or r^2^ = 0.99. Replacing *y* in the equation with our previously obtained and fitted peak position, a maximum temperature of 41 ± 10.93 °C was calculated to explain the peak drift, from 464 cm^−1^ to 465.19 cm^−1^ of the original α-quartz crystals, related to the increase in temperature caused by the heating effects of Raman laser spots.

### CL spectra

As the band positions of CL emissions depend on the specific structure of the SiO_2_ polymorph and experimental conditions, this analysis enables the interpolation of lattice defect structures from quartz crystals, which are genetically more characteristic than the content of trace element impurities^45^. The CL emission maxima of natural quartz are located at 450 and 650 nm^46,47^, but their relative luminescence intensities and splitting features reflect information about their specific pressure vs. temperature conditions during quartz precipitation.

To understand the CL spectra of quartz, some elemental replacements should be considered. Si^4+^ is commonly generally replaced by Al^3+^ and the subsequent charge deficit is compensated in magmatic and hydrothermal settings^48,49^ by other substitutes, such as K^+^ and Na^+^. The prominent emission band at around 570 nm of the neoformed quartz (Fig. 8) is related to oxygen vacancies (by original high oxygen deficiency) and high content of lattice defects. Quartz with yellow CL appears exclusively in epithermal hydrothermal environments (< 250 °C) and is associated with fast crystallization patterns under oxygen deficiency conditions^45,50,51^. The two subsidiary blue emission bands indicate strong polarization along the c-axis due to the recombination of oxygen Frenkel pairs associated with oxygen vacancy (again by oxygen deficiency) and peroxy linkage, and suggests recrystallization of quartz under diagenetic to low-grade metamorphic conditions^47,52^. A distinct blue-green emission at ~ 480–500 nm commonly reflects alkali-compensated trace-element centres in the quartz structure^52,53^, and is linked to both pegmatite, hydrothermal and metamorphic quartz^54^. Finally, patchy distribution of different luminescence spots suggests heterogeneous distribution of related quartz impurities.

### Taphonomic bias controlled by hydrothermalism

**Fig. 9:**
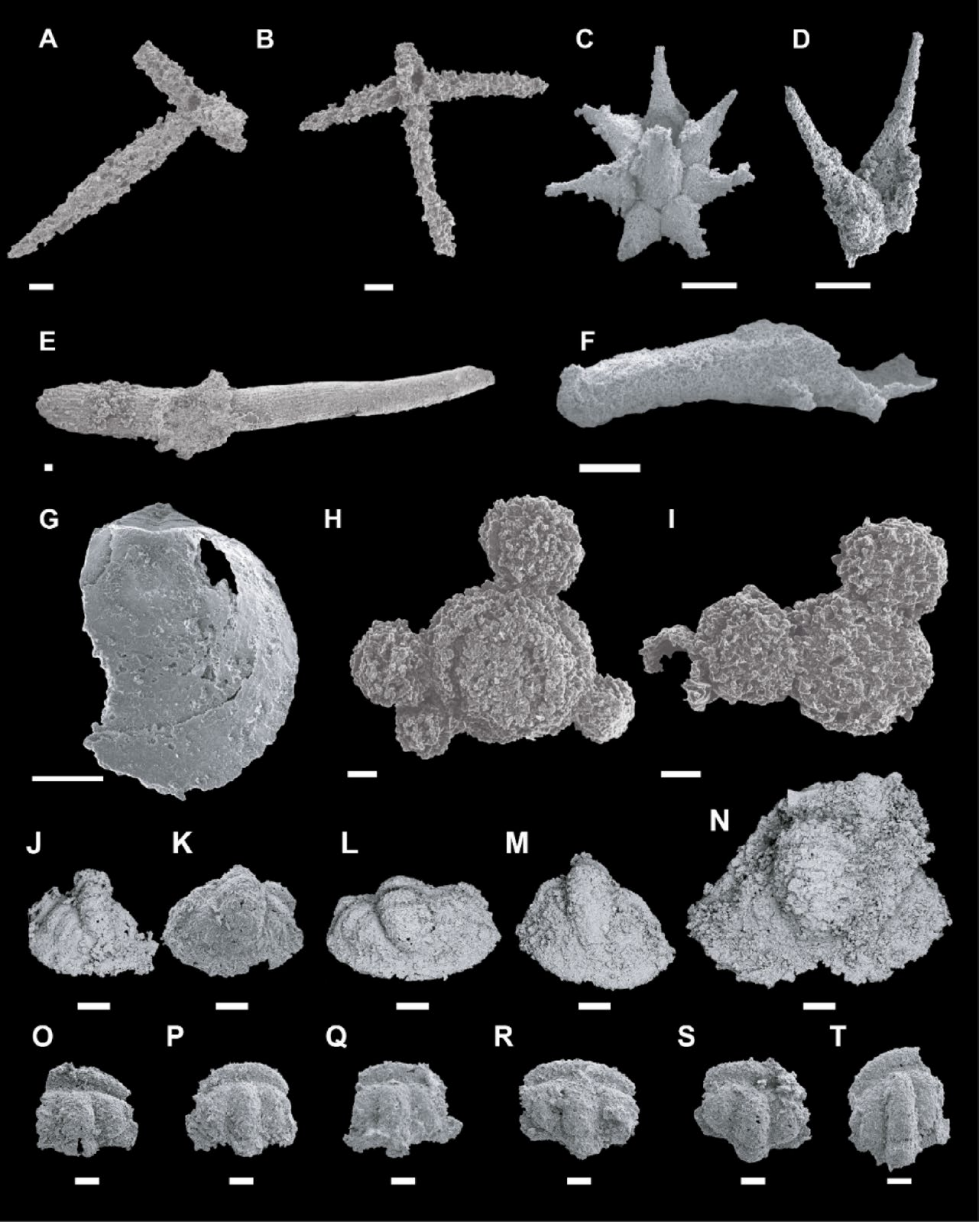
Selection of silicified skeletonized microfossils from the partly silicified limestone interbeds of the Valdemiedes Formation in the vicinity of Mesones de Isuela village. (**A**–**B**) Hexactinellid sponge spicules. (**C**–**D**) Chancelloriid specimens with partly articulated sclerites. (**E**) Fragmented librigena. (**F**) Hyolithid conch. (**G**) Disarticulated valve of linguliformean brachiopod. (**H**–**I**) Ventral and dorsal views of two psammosphaerid clusters, the former exhibiting crude concentric growth lines. (**J**–**N**) Disarticulated pygidial sclerites of ellipsocephalid trilobites. (**O**–**T**) Disarticulated cranidial sclerites of ellipsocephalid trilobites. Scale bars: A‒B, E, H‒I = 100 μm, C = 500 μm, D, F‒G, J‒T = 200 μm.

As reported above, the insolubility of silica to acetic acid dissolution favours the extraction of silicified microfossils^55,56^. As a result of etching, the limestone interbeds of the Valdemiedes Formation yields permineralized remains of a diverse assemblage of skeletonized microfossils that comprise trilobite larvae, sponge spicules and chancelloriid sclerites, echinoderm holdfasts, linguliformean brachiopod valves, and psammosphaerids and serpulids encrusting disarticulated sclerites (Fig. 9A‒T). Originally siliceous sponge (monoaxon, stauracine and hexactine) spicules are locally abundant, and their recrystallized axes are preserved as mosaics of coarse crystalline quartz. Therefore, as the silica spicules still preserve their original composition, biogenic silica, yielded by the dissolution of hexactinellid sponge spicules^57^, is not supported.

Both wholly silicified skeletons and fossiliferous remains encrusted by thin veneers of authigenic silica are commonly preserved as three-dimensional concave hyporeliefs (external moulds) and convex epireliefs (internal casts). The survival of original reliefs suggests that silicification occurred before compaction and dewatering of the thicker shale interbeds. As a result, the onset of early-diagenetic silica permineralization precludes silica sources derived from the smectite-illite transition (depth of burial ranging from 1.8 to 3.7 km) and low-grade metamorphic recrystallization of illite to muscovite, linked to a release of Si^[4+58^.

In contrast, the REE composition and the Raman and CL spectra of the micro- and megaquartz mosaics preserved in the limestone interbeds of the Valdemiedes Formation point to a hydrothermal origin of silica induced by low-pH and low-temperature waters. The hydrothermal silicification involved the texture-controlled replacement of a calcareous biota (aragonite- and calcite-walled) by silica, mainly as micro- and macrocrystalline quartz cement and chalcedony (fine-grained fibrous quartz). Fissuring and hydrothermal silicification generated secondary networks of porosity and permeability. Its related paragenesis was dominated by quartz, chalcedony and chlorite, with a variety of crystal sizes and textures that reflects stepwise phases of silicification. Chalcedony currently precipitates under the influence of slightly saturated fluids at relatively low temperatures (< 120 °C)^59^.

**Fig. 10:**
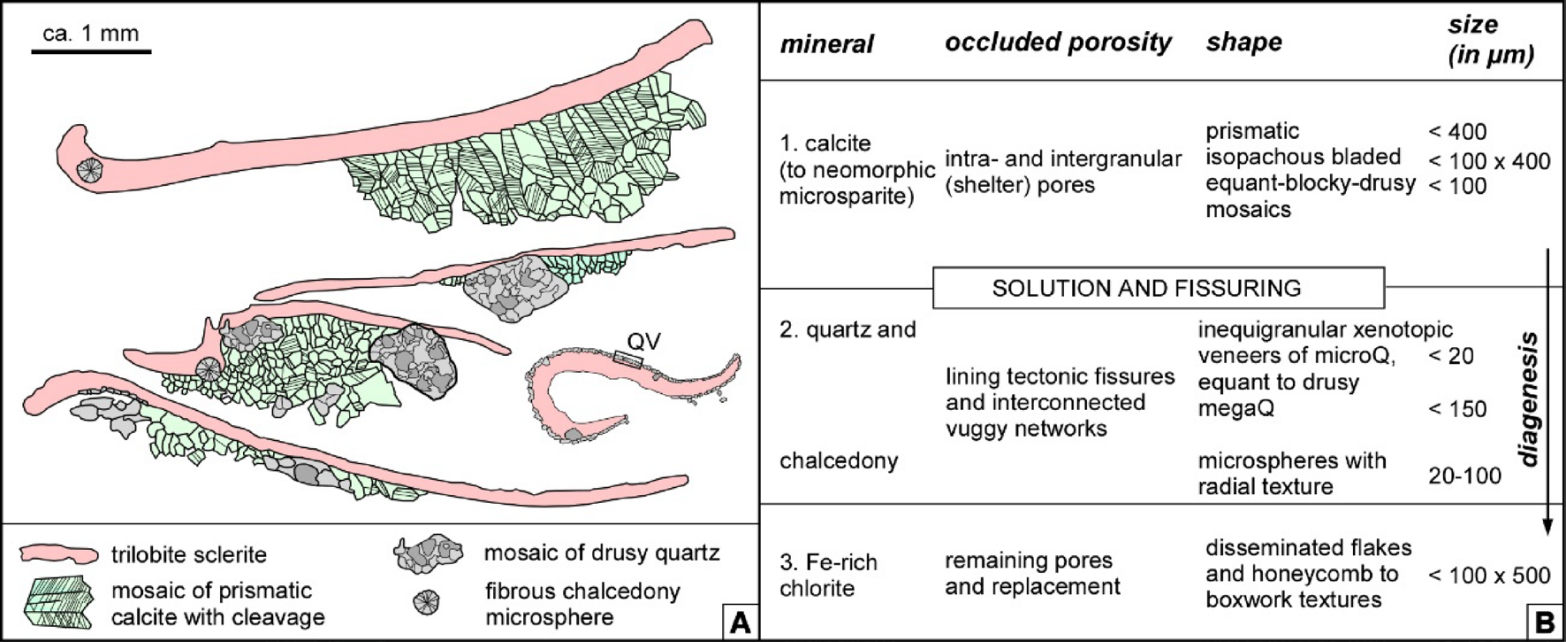
Summary of diagenetic processes recorded in the partly silicified limestones of the Valdemiedes Formation. (**A**) Sketch showing crosscutting relationships of early-diagenetic processes affecting the trilobite-rich wackestone to packstone interbeds of the Valdemiedes Formation; QV, microquartz veneer. (**B**) Idealized framework of early-diagenetic processes.

Synsedimentary faults and associated breccias linked to the Valdemiedes/Mansilla contact are not distinct, but former chronostratigraphically controlled analyses of sharp changes in sedimentation rate allowed the recognition of a major tectonic breakdown at this lithostratigraphic contact^19^. These tectonic perturbations may be reflected by the preservation of the aforementioned quartz vein stockworks associated with the analysed vuggy porosity occluded with mosaics of quartz (Fig. 10). Migrating fluid flows would have been strongly influenced by fracture systems, and laterally migrated preferentially along the shelly limestone interbeds of the shale-dominant Valdemiedes Formation^60^. The vertical lithological heterogeneity between porous limestone and shale interbeds would have favoured the lateral fluid migration of silica fluids in this mixed (carbonate-siliciclastic) formation.

### Dissolved silica sources and precipitation processes in Cambrian times

In modern oceans, dissolved silica is mostly removed from seawater through biomineralization, but, in absence of siliceous biota, the Precambrian Si cycle was dominated by (i) inorganic precipitation from saturated to nearly saturated waters^61,62^ (e.g., low-pH microenvironments favoring CaCO_3_ dissolution and SiO_2_ precipitation^63^, (ii) hydrothermal activity^64,65^, (iii) intensive terrigenous input^66^, and (iv) Si release during the smectite to illite dissolution-precipitation reaction^67^. After the Cambrian Explosion, new potential sources of oceanic dissolved silica developed. Direct abiotic precipitation processes of this widespread chert deposition includes precipitation from ambient saturated seawater^68^, secondary replacement of carbonate precursors during early or late diagenesis, as well as microbial induced sequestration (‘death mask’ preservation^3,58^ or biogenic sequestration by silica-secreting organisms like sponges and radiolarians^69^. However, during diagenetic and metamorphic conditions, pristine preservation of silicified fossils was subsequently precluded by quartz recrystallization (neomorphism). Crystal regrowth, under conditions of microstructural stress and elevated temperature, is an ubiquitous process associated with orogenesis. Hence, this case study from the Iberian Chains also provides a combination of analytical data to reconstruct the diagenetic and metamorphic effects recorded in a Cambrian hydrothermal vein stockwork from a sedimentary basin that was subsequently affected by the Variscan and Alpine orogenies.

## Conclusions

The traditional ‘lower‒middle Cambrian’ transition of the Iberian Chains is recognized within the limestone/shale alternations of the Valdemiedes Formation. Its biodiversity patterns are dramatically biased by selective silicification: macrofossil and thin-section identification from limestone and shale interbeds display variable abundances of trilobites and linguliformean brachiopods, whereas acid etching of partly silicified limestone interbeds allows identification of trilobite larvae, chancelloriids and sponge spicules, accompanied by locally abundant calcite- and phosphate-walled brachiopods, echinoderm holdfasts, and psammosphaerids and serpulids encrusting disarticulated sclerites. Acid etching allowed the identification of higher biodiversity patterns than those yielded by interbedded shales and non-silicified limestones. However, silica neomorphism destroyed ultra- and microscopic details. Fossil preservation as mosaics of micro- and macroquartz and chalcedony precludes their taxonomic assignment at the species level.

The source of silica was neither biogenically derived from dissolution of siliceous sponges nor related to the deep-burial smectite-illite transition. In contrast, the REE composition of micro- and megaquartz mosaics (chondrite-normalized REE, gull-wing patterns with a distinct negative Eu anomaly) and CL spectra (with a prominent yellow emission band at 570 nm) point to a hydrothermal origin. The Eu/Eu* ratio and presence of chalcedony reflects the circulation of slightly saturated aqueous solutions at relatively low temperatures (< 120 °C), through penecontemporaneous fissures that served as conduits for silica fluids, and were sealed (so postdated) by the overlying Mansilla Formation.

## Samples and methods

About 40 carbonate samples from the Cambrian Series 2‒Miaolingian transition (traditional ‘lower‒middle Cambrian’ transition) of the Valdemiedes Formation, in the vicinity of Mesones de Isuela, eastern Iberian Chain, were prepared for geochemical analysis, petrographic examination and acid etching. Transmitted and reflected light microscopy, scanning electron microscopy (SEM) including back-scattered electron detector (BSE) and energy-dispersive X-ray analyzer (EDX) were used for recognition of silica minerals (microquartz, macroquartz and chalcedony) in the Museo Nacional de Ciencias Naturales (MNCN), Madrid. SEM analysis was made by using a JEOL JSM-6400 fitted with an Oxford Instruments D6679 detector. Energy-dispersive X-ray (EDS) analyses and BSE imaging were obtained by SEM with accelerating voltage 20 kV, beam current 1–2 nÅ, and a counting interval of 50 s; error of analytical results reaches ± 5–7%.

Cathodoluminescence (CL)-SEM spectra were measured under the following conditions: wavelength calibration by an Hg-halogen lamp, spot diameter 30 μm, measuring time 5 s. Irradiation experiments allowed to document the behaviour of quartz crystals under electron bombardment. Samples were irradiated 5 min under constant conditions (14 kV, 0.2 mA) and spectra were measured initially and after every 1 min. The evaluation of time-dependent spectral CL measurements yielded information about the stable or transient behaviour under the electron beam and was essential for the identification of luminescence-active defect centres.

The Raman spectroscopy technique (confocal Raman microscopy, Termo Fisher DXR spectrograph, Waltham, MA, USA) of MNCN was used to characterize the composition of micro- and macroquartz. The light at 523 nm of a frequency doubled Nd: YVO4 DPSS solid laser (maximum power 30 mW) was used for excitation. Spectral data were analyzed with Termo Scientifc OMNIC Series Software and Spectragryph Analytical Software^70^.

Limestone samples (1 kg) were etched with ca. 10% acetic acid to release a mixture of variably silicified specimens from the carbonate matrix. Specimens were picked up from residues under stereomicroscope and observed and pictured with SEM at MNCN. Finally, geochemical data of etched silicified microfossils are based on 8 samples. REE were analyzed using X-ray fluorescence and inductively coupled plasma mass spectrometry (ICP-MS) (Bruker Aurora Elite spectrometer, Billerica, MA, USA) at the Complutense University, Madrid, with a sensitivity of 400,000 cps/ppb in normal mode. Eu and Ce anomalies were calculated, normalized to average chondrites), as Eu/Eu* = Eu_N_/0.5(Sm_N_+Gd_N_)^71^ and Ce/Ce* = Ce_N_ × Nd_N_/Pr_N_^2^^72^.

## Data Availability

All relevant data are in the manuscript.
